# Female Gender Remains an Independent Risk Factor for Poor Outcome after Acute Nontraumatic Intracerebral Hemorrhage

**DOI:** 10.1155/2013/219097

**Published:** 2013-09-05

**Authors:** Latha Ganti, Anunaya Jain, Neeraja Yerragondu, Minal Jain, M. Fernanda Bellolio, Rachel M. Gilmore, Alejandro Rabinstein

**Affiliations:** ^1^Department of Emergency Medicine and Neurological Surgery, University of Florida, Gainesville, FL 32608, USA; ^2^Department of Emergency Medicine, Mayo Clinic College of Medicine, Rochester, MN 55905, USA; ^3^Department of Neurosurgery, University of Rochester Medical Center, Rochester, NY 14623, USA; ^4^Department of Neurology, Mayo Clinic, Rochester, MN 55905, USA

## Abstract

*Objective*. To study whether gender influences outcome after intracerebral hemorrhage (ICH). *Methods*. Cohort study of 245 consecutive adults presenting to the emergency department with spontaneous ICH from January 2006 to December 2008. Patients with subarachnoid hemorrhage, extradural hemorrhage, and recurrence of hemorrhage were excluded. *Results*. There were no differences noted between genders in stroke severity (NIHSS) at presentation, ICH volume, or intraventricular extension (IVE) of hemorrhage. Despite this, females had 1.94 times higher odds of having a bad outcome (modified Rankin score (mRs) ≥3) as compared to males (95% CI 1.12 to 3.3) and 1.84 times higher odds of early mortality (95% CI 1.02–3.33). analyzing known variables influencing mortality in ICH, the authors found that females did have higher serum glucose levels on arrival (*P* = 0.0096) and 4.2 times higher odds for a cerebellar involvement than males (95% CI 1.63–10.75). After adjusting for age, NIHSS, glucose levels, hemorrhage volume, and IVE, female gender remained an independent predictor of early mortality (*P* = 0.0127). *Conclusions*. Female gender may be an independent predictor of early mortality in ICH patients, even after adjustment for stroke severity, hemorrhage volume, IVE, serum glucose levels, and age.

## 1. Introduction

Stroke is a priority public health problem for health systems worldwide today. Each year there are nearly 795,000 individuals who suffer from a new or recurrent stroke; 10% of these are cases of intracerebral hemorrhage. Stroke is the 4th leading cause of mortality according to the latest CDC statistics. Among all strokes, the case fatality rate for hemorrhagic strokes (37-38% mortality) is the highest [1], and most survivors have poor functional outcomes.

Female gender has been recognized as an important risk factor for stroke, with NHANES reporting that women between 45 and 54 years of age were almost twice as likely to suffer from a stroke than males [2]. A greater decline was also seen in stroke-related deaths among males as compared to females between 1980 and 2005 [3].

There is a significant literature published on gender differences in outcomes of ischemic stroke. A recent review concluded that although the incidence of stroke was higher in males, females were more severely ill [4]. Internationally, it has been reported that the stroke burden is higher in females, because of a higher prestroke and poststroke disability [5–8]. This difference in disability after stroke between men and women is seen not only physically but also psychologically [9]. No published literature, however, answers the question of gender differences specifically in acute hemorrhagic stroke.

Since acute nontraumatic intracerebral hemorrhage (ICH) contributes greatly to the poststroke morbidity and mortality burden on the health system, we sought to determine gender differences in an exclusive cohort of first time hemorrhagic stroke patients presenting to our emergency department.

## 2. Methods

This was an institutional review board approved, consecutive cohort study, conducted in the emergency department (ED) of our academic institute with an annual census of 75,000 visits. All adult patients who presented with a diagnosis of spontaneous nontraumatic intracerebral hemorrhage (ICH), from January 2006 to December 2008, were eligible for inclusion in the study cohort. All pediatric patients, patients with subdural, extradural, or subarachnoid hemorrhage, and patients with a recurrence of intracerebral hemorrhage were excluded from the final cohort.

Written consent was obtained for use of medical records from patients at admission to the hospital. The medical records of these patients were reviewed by two independent abstractors for demographics, arrival characteristics, vitals, symptoms, signs, laboratory parameters on presentation, past history of risk factors, treatment, and discharge status. The stroke severity was obtained in the form of NIHSS scores, calculated on the basis of the examination notes of the neurologist on call [10, 11]. The clinical interpretation program of the GE/Marquette Mac-8 digital ECG Chart determined the intervals on the ECG. The functional outcome at discharge was noted as the modified Rankin score (mRS) at discharge. This was stratified into two categories—good outcome (mRS < 3) and bad outcome (mRS ≥ 3) [12]. The CT scans of these patients were studied to calculate the volume of hemorrhage using the ABC/2 method [13]. The topographic area affected by the hemorrhage and the intraventricular extension of the hemorrhage were also noted. A close network of primary care and a single unified hospital system, in our county, enabled us to collect data on mortality outcomes on all patients in the study. Abstractors met periodically to discuss possible ambivalent data and to ensure uniformity in data collection. The abstractors remained blinded to the research hypothesis.

Analysis was done by using JMP 8.0 SAS Institute. All categorical and continuous variables that were collected were analyzed based upon their distributions. All associations between nominal variables were analyzed using the Pearson chi-square test or Fisher's test wherever applicable. Associations between nonnormally distributed variables were analyzed using the nonparametric Kruskal Wallis test. Multivariate logistic regression was performed to adjust for potential confounding factors to evaluate the association between gender and stroke outcome. Survival analysis based on time to death for each of the genders was also performed.

## 3. Results

Of the 261 adult patients with ICH who presented to the ED, 13 declined to consent for research; for 3 patients, this was a recurrence of ICH, and hence these patients were excluded from the cohort. In the final cohort of 245 patients, there were 125 (51.0%) females (F) and 120 (49.0%) males (M). The median age for the females was 77 years (interquartile range IQR 65 to 83 years), which was significantly higher than the median age for males (median age 69 years, IQR 59 to 80 years; *P* = 0.007). Only 12.6% of the patients were <50 years of age (12.5% females, 12.8% males).

### 3.1. Arrival Characteristics

From the cohort, 57.6% females and 56.7% males were referred to our academic ED, from elsewhere (*P* = 0.883). There was also no difference (*P* = 0.46) in the proportion of females and males who were brought in by ambulances (59.2% F, 51.7% M), helicopters (28.8% F, 32.5%  M), or private vehicles (12% F, 15.8% M).

On analyzing symptoms on presentation to the ED 37.6% females presented with headache versus 29.2% males, 28.8% females presented with vomiting/nausea versus 12.5% males, 7.2% females presented with syncope versus 5.8% males, and 46.8% females presented with weakness versus 51.3% males. Further analysis revealed that females had 2.8 times higher odds of presenting with vomiting/nausea than males (95% CI 1.45 to 5.51; *P* = 0.002). There was no difference between the other symptoms with respect to gender. About 58.9% females and 34.4% males presented with left-sided weakness, and 37.5% females and 62.5% males presented with right-sided weakness (*P* = 0.002), implying that there might be a significant difference in the area of affection, which we analyzed on CT scans. A total of 3.6% females and 3.1% males had both sides affected.

We also looked at the Glasgow coma scale (GCS) evaluation of patients in our cohort. We considered anyone to have a GCS ≤ 8 to be comatose. There was no difference between the proportion of females (26.4%) and males (25%) who arrived comatose (*P* = 0.802).

### 3.2. Stroke Characteristics (Table 1)

There was also no difference in the stroke severity on arrival as defined by NIHSS between females (median NIHSS 7, IQR 2 to 23) and males (median NIHSS 8, IQR 2 to 19; *P* = 0.76).

On assessing the CT scans on arrival of these patients, we found that there was no significant difference between the volumes of hemorrhage between females (median volume 30 cc, IQR 5.2 to 135.7 cc) and males (median volume 22.9 cc, IQR 6.5 to 85.8 cc; *P* = 0.415). There was also no difference (*P* = 0.886) in the prevalence of intraventricular extension of hemorrhage between females (47.6%) and males (46.7%).

On analyzing the topographic distribution of the hemorrhage in the brain, we found that 60% of the females had a right-sided hemorrhage as compared to 41% males. Males were more likely to have a left-sided hemorrhage (56%) as compared to females (38%). These differences were statistically significant (*P* = 0.015). The odds of having a cerebellar hemorrhage were 4.2 times higher in females than males (95% CI 1.63 to 10.75; *P* = 0.002). This association remained significant even after controlling for nausea/vomiting as the latter has been reported to be associated with higher incidence of cerebellar hemorrhage (odds ratio 3.42, 95% CI 1.16 to 10.04, *P* = 0.02). No other associations between area affected by hemorrhage and gender reached statistical significance.

### 3.3. Past Medical History (Table 2)

We compared history of ischemic stroke, coronary artery disease, hypertension, diabetes mellitus, dyslipidemia, atrial fibrillation, cancer, coagulation abnormalities, seizures, head trauma, smoking, and cessation of smoking between the genders. Females had 0.51 times odds for having coronary artery disease (*P* = 0.024) as compared to males. Also, only 30.4% females from the cohort had a past history of smoking as compared to 61.7% males (odds 0.27, 95% CI 0.16 to 0.46; *P* < 0.0001). There was, however, no difference between the proportions of females (25.6%) and males (36.5%) who stopped smoking (*P* = 0.243). Associations between past history of other diseases and gender did not reach statistical significance.

On analyzing past medication use, there was also no statistically significant difference between the class of antihypertensive medication, anticoagulants, antiplatelets, steroids, and statins (all of which are known to influence ICH) used by males and females.

### 3.4. Vitals and Laboratory Parameters (Also See Table 3)

There was no difference in the skin temperature (*P* = 0.467), heart rate (*P* = 0.725), and systolic blood pressure (*P* = 0.271) recorded on arrival to the ED between males and females. The women, however, did have a lower diastolic blood pressure on arrival (median 81 mmHg, IQR 71 to 95 mmHg) when compared to the men (median 87 mmHg, IQR 75 to 102 mmHg; *P* = 0.011).

On analyzing the ECG intervals (PR, QRS, and QTc), we found a significantly lower QRS interval in females (median 90 ms, IQR 82 to 98 ms) when compared to males (median 94 ms, IQR 88 to 106 ms; *P* = 0.0125). There was no difference between the genders in relation to the other ECG intervals.

On analyzing hemogram characteristics, we noted that females had lower hemoglobin (median 13.2 mg/dL, IQR 12.3 to 14.1) as compared to males (median 14.1 mg/dL, IQR 12.7 to 15.3 mg/dL; *P* < 0.0001), higher leukocyte counts (median 10.7 × 10^3^/cc for females versus 9.4 × 10^3^/cc for males; *P* = 0.039), and higher platelet counts (median 244 × 10^3^/cc for females versus 223 × 10^3^/cc; *P* = 0.0031), but all these were within normal limits. There were no differences between the genders with regard to the coagulation parameters INR (*P* = 0.532) and aPTT (*P* = 0.096).

Females also had higher total cholesterol levels (median 199 mg/dL IQR 174 to 211 mg/dL) when compared to males (median 168 mg/dL, IQR 146 to 208 mg/dL; *P* = 0.033). Women also had higher measured blood glucose on arrival to ED (median 146 mg/dL IQR 115 to 191 mg/dL) when compared to men (median 129 mg/dL, IQR 106 to 157 mg/dL; *P* = 0.0096).

There were also statistically significant differences that were noted between serum sodium (*P* = 0.0386), serum potassium (*P* = 0.0045), and serum creatinine (*P* < 0.0001) levels between females and males, with females having lesser values than males. However, these differences were not clinically significant.

### 3.5. Poststroke Characteristics

A total of 12.8% females had a craniotomy/surgery as compared to 10.8% of the male subjects, and nearly 10.4% females had an extraventricular drainage (EVD) as compared to 8.3% males. However, there was no difference in proportion of the females undergoing surgery/craniotomy and EVD as compared to the males (craniotomy/surgery *P* = 0.62, IVD, *P* = 0.58).

### 3.6. Stroke Outcomes (Also See Table 4)

There was no difference in the median length of hospital stay between females (median 4 days, IQR 2 to 10 days) and males (median 5 days, IQR 3 to 13 days; *P* = 0.179).

Females had a worse functional outcome on discharge (median mRS 4, IQR 2 to 6) when compared to males (median mRS 4, IQR 0 to 5; *P* = 0.0062). Females had 1.94 times the odds of having a poor outcome as compared to males (95% CI 1.12 to 3.3).

There were also more deaths within 7 days in the female subset (*n* = 38, 30.4% patients) as compared to the males (*n* = 23, 19.2% patients). The odds of dying within 7 days were 1.84 times higher in females than in males (95% CI 1.02 to 3.33; *P* = 0.0421).

### 3.7. DNR Orders

New DNR orders were instituted over a period of 1–13 days from the date of admission. Overall, 62 (49.6%) females had DNR orders versus 36 (30%) of males.

Despite having no differences between the stroke severity, volume of hemorrhage, and past history of diseases, females were also more likely to have DNR (do not resuscitate) orders instituted after admission to the hospital with ICH (odds ratio 2.33, 95% CI 1.37 to 2.97; *P* = 0.0016). The reasons for this female predilection were not studied.

We created a nonearly DNR subset analysis for our cohort. To create this subset, we first made a subcohort of all patients who had DNR orders instituted within 24 hours (*n* = 44). In this subcohort, we excluded any patients who received any operative or interventional procedures after arrival to the ED (*n* = 2), because it was assumed that these patients were only made DNR after significant interventional albeit futile, efforts. The final subcohort (*n* = 42; females 28, males 14) was then omitted from our initial cohort of 245 patients. This final subset of nonearly DNR patients (*n* = 203) consisted of 97 females (47.8%) and 106 males (52.2%). On analysis of association of gender with early mortality, we still found that females were 2.6 times (OR) more likely to die within 7 days of presentation with ICH than males (95% CI 1.3 to 5.4; *P* = 0.006).

### 3.8. Multivariate and Survival Analysis (Also See Table 5 and Figure 1)

It is a known fact that increasing age, stroke severity, cerebellar hemorrhage, and intraventricular extension of hemorrhage are associated with poor outcome and death after ICH. We ran a logistic fit incorporating all these variables in a multivariate analysis and found that female gender remained an independent predictor of poor outcome at discharge (adjusted OR = 2.30, 95% CI 1.04 to 5.25; *P* = 0.0398) and death within 7 days of ICH (adjusted OR 3.03, 95% 1.24 to 7.79; *P* = 0.0144). The adjusted OR for mRs for males was 0.44 (95% CI 0.19 to 0.96) and for death within 7 days was 0.33 (95% CI 0.13 to 0.81).

**Figure 1:**
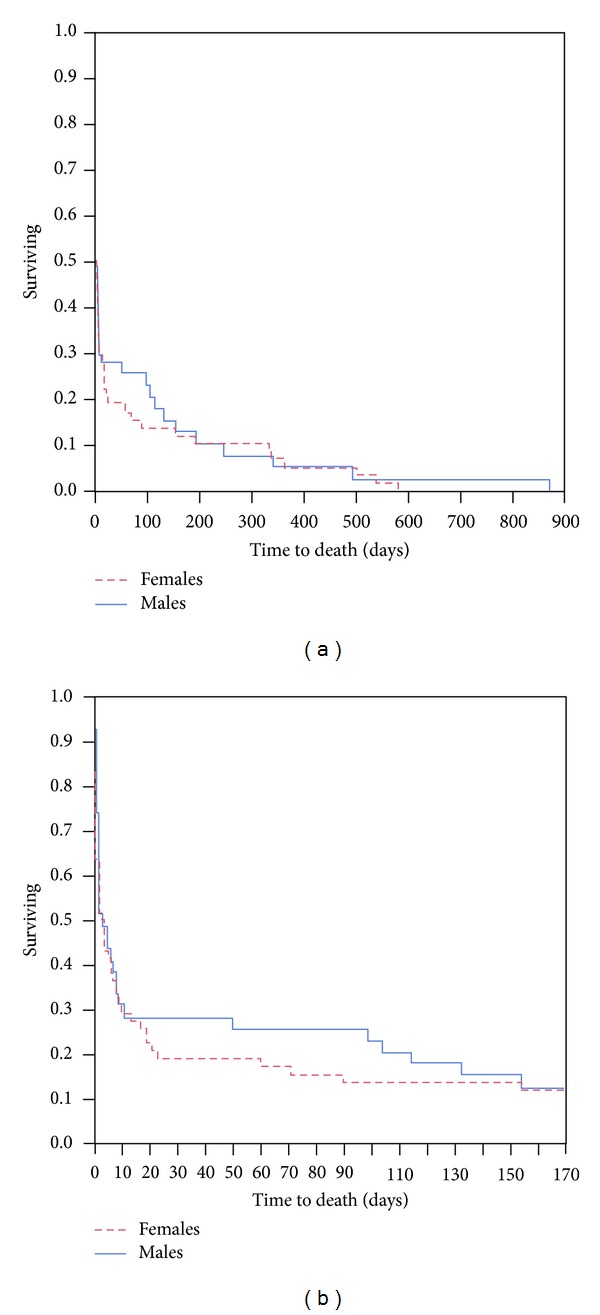
Survival curves for males and females with respect to time to death in days after intracerebral hemorrhage.

We also ran a survival analysis based on time to death for each of the genders. The curves were near similar and the statistical analysis of difference did not reach significance on the Wilcoxon test (*P* = 0.4403). A closer look at the curves did show a clear separation between the mortality trends among females and males between 10 and 150 days after ICH (Figure 1).

## 4. Discussion

In this research, we have reported important differences between males and females who presented with first time intracerebral hemorrhage (ICH). Our demographic finding of women being older than men at the time of ICH concurs with the findings of Roquer et al. who reported that women were on an average 6 years older than men at the time of stroke [6].

Although there were no differences between the modes of arrival of patients for both genders, or their state of consciousness and orientation (measured by GCS), we did find that women were more likely to have nausea/vomiting as a symptom on presentation. Investigators have previously reported some differences in the presentation symptoms between males and females with stroke, with regard to ability to walk [14], aphasia, visual field disturbances, dysphagia [6], and coma [5]. We did not find similar differences in our cohort. The most important difference that we noted however, was that women were more likely to be affected in the right hemisphere, causing left-sided weakness. This predilection for right hemispheric affection in ICH in females is previously unreported. We also noted that though the volume of hemorrhage was similar in both genders, females had nearly 4 times the odds to have a cerebellarhemorrhage when compared to males. This actually steps away from previous reports on ischemic stroke, which state that infra-tentorial strokes are less prevalent in females [7, 14].

Previous experience with subarachnoid hemorrhage (SAH) patients has revealed that females with SAH may be more prone to hypokalemia and QT prolongation [15]. A similar trend was also seen for the females in our cohort. These patients had lower potassium, sodium, and creatinine levels. Although statistically significant, the difference, however, was not clinically significant. Females also had lower hemoglobin, higher leukocyte, and platelet counts as compared to males, but again these were all within normal limits. Women did have higher glucose levels on arrival to the emergency department, although there was no difference in the prevalence of diabetes mellitus between males and females. It has been reported in the past that hyperglycemia on presentation can lead to worse early outcome in patients with spontaneous ICH [16].

We concentrated on studying the early mortality outcomes for our cohort, as we wanted to negate influences of complications that arise due to the morbidity and disability after stroke. We found that, despite having similar premorbid status, similar prior medication use, similar hemorrhage volumes and frequency of intraventricular extension, women still experienced higher 7-day mortality post ICH. The higher odds of cerebellar ICH and hyperglycemia at presentation, could be contributory factors to this phenomenon. It is known that hyperglycemia early on in the course of spontaneous ICH does influence the early outcome in these patients [16]. The long-term mortality experience for males and females was, however, similar, as we saw on the survival curves.

At discharge from the hospital, females were almost twice as likely as males to have bad functional outcome. This association held true even after adjusting for predictors of worse outcome in a multivariate model.

Higher inflammatory response in hemorrhagic stroke has been associated with poor outcomes [17, 18]; studies have reported that females tend to have more robust inflammatory response in some diseases [19]. This could have led to poor outcome in females, although we were not able to capture data regarding inflammatory measures such as midline shift, perihematomal edema, or herniation.

There are differing viewpoints when it comes to female gender and stroke. For example, it has been reported that progesterone and estrogen may be important neuroprotective agents in ischemic stroke [20–23]. Research has suggested that estrogens may serve this purpose by a way of stimulating growth factor supply, attenuating inflammatory processes, free radical scavenging, stimulation of intrinsic antiapoptotic pathways, and other interactions with intrinsic cell-cell pathways [24–26]. Some researchers have also suggested the mitochondria as a possible site of estrogen-mediated, neuronal survival [27]. Other studies have reported that women experience worse functional outcome and early mortality after ischemic stroke [8, 28]. Still others, however have credited the female gender for conferring a survival advantage after ischemic stroke [29], consistent with the notion that female sex hormones are candidates for neuroprotective therapy.

Some researchers have reported that gender differences in outcome are not seen in hemorrhagic stroke [30]. Our study reports the first comprehensive gender differences in a cohort of exclusively first time hemorrhagic stroke patients. The significant differences outcomes and early mortality, with female sex driving bad outcome, may indicate that unlike in ischemic stroke, female sex hormones might not present a survival advantage in ICH. This may be due to intrinsic differences in the neuronal damage mechanisms in ICH and ischemic strokes, which need to be studied extensively in the near future.

## 5. Limitations

We noted that female patients in our cohort were more likely to have DNR orders instituted after admission for ICH, despite the similarities to males on premorbid conditions, stroke severity and hemorrhage volumes. The variation in the use of DNR orders has been studied extensively including differences by gender, race, insurance status, patient preference, hospital, and specialty [31, 32]. However, we did not collect those variables in our study. It might be suggested that DNR orders could have influenced the outcomes and could have been an important confounder in our study. By doing the subset analysis, wherein we excluded patients who had early DNR orders, a surrogate for no interventions, we believe that we tried to get an understanding of this influence. If anything, the female gender became more strongly associated with early mortality in this subcohort of nonearly DNR patients (odds ratio increased from 1.84 to 2.33).

For convenience, we looked at functional outcome at the hospital discharge, rather than at a fixed time the point from point of onset of ICH itself. This could have influenced our assessment of mRS, though we did not notice any statistically significant difference in hospital length of stay between the two genders. In addition to this, we did not report long-term outcome for our patients. Although prospective large-scale stroke trials typically assess outcome at 3–6 months, due to the unavailability of data and the retrospective nature of our study, we were unable to do so. We hope to address this limitation in future prospective studies.

We also could not assess the effects of hematoma expansion in our cohort, as the initial CT scan was done at different time points from onset of ICH, and results of repeated CT scan were not looked at. It is known that hematoma expansion can continue for up to 48 hours after stroke [33]; hence, we may have overestimated or underestimated the volume of hemorrhage in our patients.

Referral of a large subset of patients from other hospitals and initial treatment given at other sites could have influenced our assessment of initial GCS.

Since only 12.5% of our female subset was below the age of 50 years (the median age for menopause) [34], we might not be seeing the protective effects of estrogen on our cohort, which may truly hold true.

We also did not look at specific causes of mortality in our cohort of patients. But since we are reporting early mortality after ICH, we have fair reason to believe that death within 7 days was primarily related to the ICH itself.

## 6. Conclusion

We have described important gender differences in patients with first episode of ICH. We have concluded that unlike in ischemic stroke females are more likely to experience cerebellar hemorrhagic strokes, than males. Also, we have reported a greater inclination for females to experience a right hemispheric ICH than males, which could influence functional recovery and mortality outcomes. We found that females have nearly twice the odds to die within 7 days after an ICH as compared to males. Female gender is an independent predictor of early mortality and bad functional outcome at hospital discharge, after adjusting for age, stroke severity, volume of hemorrhage, and intraventricular extension of hemorrhage, all of which are known to predict worse outcome.

## Figures and Tables

**Table 1:** Gender differences in stroke characteristics.

Characteristic	Females	Males	*P* value
NIHSS			
Median	7	8	0.759
IQR	2–23	2–19	
Volume			
Median	30 cc	22.9 cc	0.415
IQR	5.2–134.7 cc	6.5–85.8 cc	
Intraventricular hemorrhage	59 (47.6%)	56 (46.7%)	0.886
Affected hemisphere			
Right	71 (59.7%)	47 (40.9%)	0.0146
Left	47 (37.8%)	65 (56.5%)	
Bilateral	3 (2.5%)	3 (2.6%)	
Topographical distr.			
Frontal	21 (17.8%)	25 (22.7%)	0.354
Temporal	21 (17.8%)	12 (10.9%)	0.139
Parietal	25 (21.2%)	15 (13.7%)	0.134
Occipital	7 (5.9%)	7 (6.4%)	0.892
Midbrain	3 (2.5%)	0	0.248
Pons	6 (5.1%)	3 (2.8%)	0.501
Cerebellum	23 (19.5%)	6 (5.5%)	0.0015
Basal Ganglia	18 (15.3%)	27 (24.5%)	0.078
Internal Capsule	1 (1%)	9 (8.2%)	0.008
Thalamus	19 (16.1%)	18 (16.4%)	0.957
Putamen	2 (1.7%)	6 (5.5%)	0.159

**Table 2:** Gender differences in premorbidity of patients with ICH.

Characteristic	Females	Males	*P* value
Hypertension	90 (72%)	86 (71.7%)	0.954
Coronary artery disease	23 (18.4%)	37 (30.8%)	0.0237
Diabetes mellitus	23 (18.4%)	24 (20%)	0.75
Ischemic stroke	17 (13.6%)	12 (10%)	0.383
Dyslipidemia	53 (42.4%)	46 (38.3%)	0.517
Atrial fibrillation	9 (7.3%)	13 (11.1%)	0.299
Coagulation disorders	4 (3.2%)	0	0.272
Head trauma	5 (4%)	5 (4.1%)	0.947
Seizures	9 (7.2%)	17 (14.2%)	0.077
Cancer	19 (15.2%)	25 (20.9%)	0.114
Smokers	38 (30.4%)	74 (61.7%)	<0.0001
Stopped smoking	29 (74.4%)	47 (63.5%)	0.243

**Table 3:** Gender differences in vitals and laboratory parameters after ICH.

Characteristic	Females	Males	*P* value
Temperature			
Heart rate	78 (70–91)	78 (65–88)	0.725
Blood pressure			
Systolic	161 (144–184)	155 (134–178)	0.271
Diastolic	81 (71–75)	87 (75–102)	0.0107
ECG			
PR interval	168 (148–190)	168 (152–186)	0.921
QRS duration	90 (82–98)	94 (88–106)	0.0125
QTc interval	451 (427–476)	437 (425–465)	0.059
Hemogram			
Hemoglobin	13.2 (12.3–14.1)	14.1 (12.7–15.3)	<0.0001
Leukocytes	10.7 (7.9–13.4)	9.4 (6.9–12.2)	0.0389
Platelets	244 (209–294)	223 (177–267)	0.0031
INR	1 (0.9–1.5)	1 (1–1.1)	0.532
aPTT	25 (23–28.6)	26 (23.7–29)	0.097
Electrolytes			
Na+	137 (135–140)	138 (126–140)	0.0387
K+	3.9 (3.5–4.2)	4 (3.8–4.4)	0.0046
Cl−	102 (98–105)	103 (100–106)	0.059
Mg++	2.1 (1.9–2.2)	2.1 (1.9–2.2)	0.394
Creatinine	0.8 (0.6–1.1)	1 (0.8–1.1)	<0.0001
Blood urea nitrogen	15 (12–20)	17 (13–22)	0.064
Cholesterol	199 (174–211)	168 (146–208)	0.0334
Glucose	146 (115–191)	129 (106–157)	0.0096

**Table 4:** Unadjusted and odds ratio with 95% CI of having poor outcome in males and females after ICH.

Outcome variables	Females		Males	
	Unadjusted OR (95% CI)	Adjusted OR* (95% CI)	Unadjusted OR (95% CI)	Adjusted OR*(95% CI)
mRs	1.94 (1.12–3.3)	2.30 (1.04–5.25)	0.52 (0.30–0.89)	0.44 (0.19–0.96)
Death (<7 days)	1.84 (1.02–3.33)	3.03 (1.24–7.79)	0.54 (0.30–0.98)	0.33 (0.13–0.81)

*After adjusting for log age, log NIHSS, cerebellar hemorrhage, and IVH extension.

**Table 5:** Logistic regression model for poor outcome.

Outcome variables	mRs (≥3)		Death (<7 days)	
	*P* value	95% CI	*P* value	95% CI
Female gender	0.0431	1.04 to 5.25	0.0173	1.24 to 7.79
Log age	0.0012	6.42 to 1384.3	0.0735	0.79 to 246.3
Log NIHSS	<0.0001	31.12 to 687.2	<0.0001	73.38 to 4815.96
Cerebellar hemorrhage	0.556	0.23 to 2.29	0.94	0.24 to 3.55
IVH extension	0.0023	1.64 to 8.96	0.0002	2.38 to 15.25
